# Editorial: Hearing loss rehabilitation and higher-order auditory and cognitive processing

**DOI:** 10.3389/fneur.2023.1257554

**Published:** 2023-07-25

**Authors:** James G. Naples, Helen Henshaw, Piers Dawes, Aaron C. Moberly

**Affiliations:** ^1^Harvard Medical School, Beth Israel Deaconess Medical Center, Boston, MA, United States; ^2^Hearing Sciences, Mental Health and Clinical Neurosciences, School of Medicine, University of Nottingham, Nottingham, United Kingdom; ^3^National Institute for Health and Care Research, Nottingham Biomedical Research Centre, Nottingham, United Kingdom; ^4^Centre for Hearing Research, School of Health and Rehabilitation Sciences, University of Queensland, St. Lucia, QLD, Australia; ^5^Vanderbilt University Medical Center, Nashville, TN, United States

**Keywords:** hearing loss, cognitive neuroscience, auditory neuroscience, cochlear implantation, central auditory processing

The associations between hearing loss and cognition are complex. Over the last decade, our understanding of some of the underlying mechanisms that contribute to this association have emerged. Additionally, more recent research has focused on the role of auditory rehabilitation on cognitive function. It is an exciting time to be a part of the interface of otologic medicine and surgery and cognitive hearing science research because of the rate at which knowledge is amassing in these disciplines. Each new idea represents a step forward and builds upon prior work. The breadth of this research is vast and involves a number of different research and clinical specialists that not only brings readers from the bench to the bedside, but also introduces them to ideas from surgeons to psychologists. With the range of specialists active in research of the associations of hearing loss and cognition, the field will likely continue to expand and evolve in a positive way. In this article collection we aim to highlight some of the ongoing research by demonstrating the breadth of the work.

With fifteen articles in the Research Topic and ninety-five authors, the included authors represent multiple countries spanning the globe. Beyond the diversity of our authors, the topics being researched are just as diverse, as are the types of research reported, from basic experimental research to applied science studies of patient and public involvement. Our authors present work that studies the association of hearing loss and cognition across the life span. Jamsek et al. and Zhou et al. start by presenting research that explores the associations between executive function and cortical responses in children with hearing loss. van Wieringen et al. studied how sensorimotor and cognitive functions are coupled in mid- to late-adulthood. Jiang et al. evaluate the associations of audiometric hearing and speech-in-noise performance with cognitive decline in the aging population (>60 years old), and Burleson et al. explore the cognitive-linguistic abilities that contribute to perceptual restoration of degraded speech. Slade et al. present a meta-analysis of the impact of age-related hearing loss on structural neuroanatomy.

In addition to the impact of age on the association of hearing and cognition, the type of hearing loss and mode of intervention were explored in research for this article collection. Qiao et al. demonstrated central reorganization patterns in patients with single-sided deafness, while others explore bilateral hearing loss patterns. The impact of hearing aids (Moradi et al.) and cochlear implants (Völter, Götze et al.; Beckers et al.) are assessed in various studies as well, along with the potential impact of cognition on device programming (Windle et al.). Evolving forms of assessment of hearing loss and cognition in clinical populations are reported by Tarawneh et al. and Völter, Fricke et al.. Mathias et al. offer research that introduces the notion of genetic factors influencing the associations between cognition and hearing loss. Finally, Broome et al. explore patient perceptions of cognitive testing within the adult hearing service model.

This article collection covers incredible breadth and depth in the field of cognitive hearing science. As the fields of otology and cognitive hearing science continue to evolve and expand, so too will the collaborations that exist among clinicians and researchers. While our article collection brings clarity to a number of complicated questions related to hearing loss and cognition, it simultaneously blurs the distinction between surgeon, psychologist, and scientist. Similarly, the once clear-cut roles of the ear and the brain are becoming cloudy. The work we present to readers in this article collection represents a solid foundation on which future research can be established. We are fortunate to be involved in contributing to this foundation, and we are eager to see how this future evolves.

## Author contributions

All authors listed have made a substantial, direct, and intellectual contribution to the work and approved it for publication.

